# Advances in surveillance and control methods for *Aedes*-borne diseases and urban vectors: report of the International Conference, August 2024, Tanzania

**DOI:** 10.1186/s13071-025-06838-4

**Published:** 2025-06-06

**Authors:** Anne Poinsignon, Florence Fournet, Halfan S. Ngowo, Victoria Franco Martins Barreira, João Pinto, Frederic Bartumeus, Emmanuel W. Kaindoa, Vincent Corbel

**Affiliations:** 1 Infectious Diseases and Vectors: Ecology, Genetics, Evolution and Control (MIVEGEC), University of Montpellier, IRD, CNRS, Montpellier, France; 2 Department of Environmental Health and Ecological Sciences, Ifakara Health Institute, Ifakara, Tanzania; 3 Instituto de Higiene e Medicina Tropical, Global Health and Tropical Medicine (GHTM), LA-REAL, Universidade Nova de Lisboa, Lisbon, Portugal; 4 Centre d’Estudis Avançats de Blanes (CEAB)–Spanish National Research Council (CSIC), Blanes, Spain; 5 Institució Catalana de Recerca i Estudis Avançats (ICREA), Barcelona, Spain; 6 Instituto Oswaldo Cruz (IOC/Fundação Oswaldo Cruz [FIOCRUZ]), Rio de Janeiro, Brazil

**Keywords:** Arbovirus, *Aedes spp.*, *Anopheles stephensi*, Vector control and surveillance, Citizen science, Innovative tools

## Abstract

Vector-borne diseases, particularly arboviral diseases transmitted by mosquitoes (e.g. dengue, Zika and chikungunya), have (re)emerged globally with increasing prevalence and severity. Climatic and environmental changes have resulted in significant expansion of the geographical distribution of *Aedes* mosquito vectors to unprecedented levels, creating optimal conditions for their introduction and establishment in new areas, especially in Africa. The prevention of *Aedes*-borne diseases relies heavily on controlling vector populations. However, the global resurgence of dengue underscores the limitations of current vector control tools in preventing epidemics, highlighting the urgent need for affordable, scalable and community-based vector control measures to address *Aedes*-borne diseases and urban mosquito vectors (e.g. *Aedes* spp. and *Anopheles stephensi*), with the overall aim to improve public health and well-being. In this report, we summarize the main outcomes of the “International conference on advances in surveillance and control methods for *Aedes*-borne diseases and urban vectors” held in Dar es Salaam, Tanzania, 26–28 August 2024. The conference aimed to facilitate knowledge exchange, promote collaborative research and drive innovation in the surveillance and control of *Aedes*-borne diseases in Africa. Key objectives included reviewing the performance of new tools and technologies for *Aedes* control, and fostering inter-sectoral and international collaborations to strengthen public health measures against mosquito-borne diseases. The event was attended by more than 200 participants from 20 nationalities/countries and was streamed live online, with 321 virtual accesses recorded during the 3-day event.

**Graphical Abstract:**

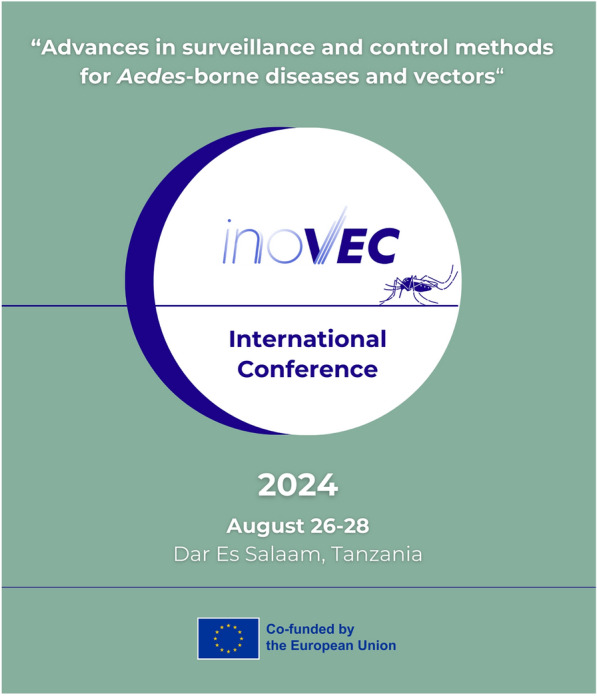

**Supplementary Information:**

The online version contains supplementary material available at 10.1186/s13071-025-06838-4.

## Background

Over the last decades, there has been a significant rise in the prevalence and severity of arboviral diseases transmitted by mosquitoes, such as dengue, Zika, chikungunya and yellow fever. Between 2000 and 2019, the WHO reported a 10-fold increase in the number of dengue cases reported worldwide, reaching 5.2 million cases in 2019 [1]. In 2023, there were over 5 million dengue cases with over 5000 related deaths reported across 80 countries/territories, making it a historically alarming year. According to the WHO, Africa ranked among the top four regions most affected by dengue, with 171,991 documented cases and 753 deaths in 2023, highlighting the urgent need to provide adequate and coordinated response to this growing threat [1].

Although the WHO has recently prequalified a second dengue vaccine, the availability of effective vaccines and treatments remains limited. Consequently, vector control continues to be the cornerstone of programs aimed at preventing and controlling *Aedes*-borne arboviral human infections. Unfortunately, the global resurgence of dengue worldwide, including Africa, highlights the limitations of current vector control tools in preventing epidemics. Beyond *Aedes* mosquitoes, Africa faces new challenges with the introduction and spread of the invasive malaria vector *Anopheles stephensi*, which has been reported in 10 countries across the continent to date [2]. Consequently, new affordable, scalable and community-based vector control measures are urgently needed to tackle *Aedes*-borne diseases (ABD) and other urban mosquito vectors to contribute to better health and human well-being.

In August 26–28, 2024, Ifakara Health Institute (IHI) and Institut de Recherche pour le Développement (IRD), with support from the European INOVEC project (grant no. 101086257) jointly organized the international conference “Advances in surveillance and control methods for *Aedes*-borne diseases and urban vectors” in Dar es Salaam, Tanzania. This conference served as a forum for exchanges within the scientific community, including scientists, students and academia as well as representatives from public health sectors, stakeholders, donor agencies, industry and international organizations engaged in controlling vector-borne diseases. The objectives were: (i) to facilitate knowledge exchange and promote collaborative research and innovation for the surveillance and control of *Aedes* and ABDs in Africa and beyond; (ii) to review the performances of new tools, approaches and technologies for urban mosquito control, identifying gaps and priorities; and (iii) to promote inter-sectoral and international collaborations and facilitate discussions for actionable outcomes for improved public health measures against mosquito borne diseases.

The conference was structured around three scientific themes: “Biology and ecology of invasive vectors”; “Integrated approaches to vector surveillance”; and “Innovation in vector control.” Each theme featured two keynote lectures and about 10 oral scientific presentations. A dedicated PhD session provided early-stage researchers with the chance to present their work and find new opportunities to stimulate their careers. Awards were given to the top three presenters. In addition, an industry plenary session enabled companies and start-ups involved in Research and Development (R&D) to present the latest advances in vector control tools and technologies. In addition, 13 posters and five turbo talks were presented by scientists, students and industry representatives on various themes. Finally, a round table open to all participants was organized to discuss the challenges and opportunities in controlling the spread of *An. stephensi* and *Aedes* spp. mosquitoes in urban settings. The meeting agenda, list of speakers, names of registered participants and abstracts are available online. This multi-sectoral conference brought together over 200 participants from 20 nationalities, and 321 views were recorded by the organizers over the 3 days of the conference.

## Welcoming addresses

The first day of the conference was opened with welcoming addresses by representatives from the Ifakara Health Institute (Honorati Masanja, Chief Executive Director), the Ministry of Health, Tanzania (Ahmad Makuwani) and the French Embassy in Tanzania (Luc Blanco, Cooperation and Cultural Action Advisor). The Chair of the conference, Emmanuel Kaindoa (IHI, Tanzania) outlined the objectives of the conference and thanked all sponsors, partners and supporting organizations that contributed to the organization of the event. All speakers emphasized the scale of the emergency caused by arboviral diseases worldwide and called for stronger commitment and international cooperation to sustain vector control and surveillance, particularly in the African continent. During this session, Raman Velayudhan, Unit Head at the Veterinary Public Health, Vector Control and Environment Unit in the Global Neglected Tropical Diseases (NTD) Program of the WHO, delivered a keynote presentation on the global dengue situation. He revealed that in 2024, over 11 million dengue cases were reported to WHO, with 102 countries experiencing active dengue transmission. Despite this alarming burden, global capacity to respond to multiple, concurrent outbreaks remains strained due to resource shortages, including limited dengue diagnostic kits for early detection, insufficiently trained clinical and vector control staff and low community awareness. The speaker emphasized that the risk of arboviruses with epidemic potential emerging and re-emerging has intensified as a global public health threat and is expected to increase in the coming years due to the global changes. The recently adopted WHO Global Arbovirus Initiative aims to optimize the use of limited resources to achieve maximum impact, particularly in areas with the highest arboviral burden and those at risk of future outbreaks [3]. Vincent Corbel (IRD/FIOCRUZ, Brazil), Co-Chair of the event, introduced the HORIZON-EUROPE INOVEC project that represents a vast pan-European, cross-sectoral and multidisciplinary network to develop, optimize and promote integrated approaches and innovative tools for the surveillance and control of mosquito vectors of emerging arboviruses. The project brings together 25 academic and non-academic institutions specialized in vector biology, mathematics, social sciences and product development. Its objectives include stimulating research, strengthening capacities, promoting career development and facilitating knowledge transfer across sectors and regions. The ultimate goal of the INOVEC project is to contribute to international efforts to improve global health and well-being by reducing the burden of vector borne diseases.

## Session 1: biology and ecology of invasive vectors

The first scientific session opened with two keynote lectures, including an insightful presentation by Yeromin P. Mlacha (IHI, Tanzania) on leveraging deep learning and remote sensing to combat mosquito-borne diseases in urban settings, with a focus on cities such as Dar es Salaam. His presentation emphasized the pivotal role of urban morphology—including housing structures, neighborhood environments and land-use patterns—in shaping mosquito-borne disease transmission. The speaker illustrated how integrating multi-scale data sources can identify high-risk areas and predict transmission patterns more effectively. These sources include household surveys to assess housing conditions and mosquito densities, geospatial surveys to capture neighborhood characteristics and drone imagery to map urban landscapes with high precision. By integrating these diverse datasets into deep learning models, researchers can more accurately identify high-risk areas and predict transmission patterns. Additionally, the incorporation of routine surveillance data enhances an understanding of mosquito vector dynamics and their environmental determinants, enabling more targeted interventions. Despite challenges such as the quality and availability of data and the need for technical capacity, this innovative methodology offers promising solutions to reduce the burden of vector-borne diseases in Africa.

Heather M. Ferguson (University of Glasgow, Scotland) delivered a compelling presentation on the “Mosquito Scotland” project, a multidisciplinary initiative addressing the growing risk of zoonotic mosquito-borne diseases in Scotland, which is driven by climate changes. While mosquito-borne pathogens, such as West Nile and Usutu viruses, are pathogens attracting increasing concerning across Europe due to the expanding range of mosquito vectors like *Culex pipiens*, surveillance efforts in the UK remain limited, with almost no data from Scotland, resulting in critical data gaps. The project adopts an integrative approach, combining entomological, pathogen and wildlife surveillance with advanced modeling to evaluate the potential for mosquito borne-disease transmission under current and projected climate scenarios. Key findings include the identification of potential mosquito vectors from nationwide mosquito surveillance at Scottish wetlands and insights from a citizen science platform, which revealed previously undocumented hotspots of *Aedes* mosquito activity. This work underscores the value of public engagement in improving preparedness for zoonotic mosquito borne-disease emergence.

Alfred J. Simfukwe (IHI, Tanzania) presented findings from a study assessing the risk of mosquito-borne diseases along the Tanzania-Zambia Railway (TAZARA). Global trade and travel have accelerated the spread of invasive disease vector mosquitoes, with serious implications for human health [4]. The railway crosses diverse ecological zones that facilitate mosquito proliferation, and the study found that *Ae. aegypti* accounted for 21% of total mosquito catches, with activity observed on trains during both daytime (75.7%) and nighttime (60.1%). Habitat analysis identified stagnant water bodies as the predominant breeding sites around the train stations, comprising 71.2% of the total. A passenger survey assessing perceptions and awareness of mosquito-biting risks highlighted the low use of protective measures during travel, with only 10% of travelers reported to have used repellents, and most relying on ineffective methods like swatting. The study underscores the need for integrated mosquito management strategies, including monitoring and public health education, to reduce the risk of mosquito-borne diseases in the region.

Alphonse K. Konkon (Centre de Recherche Entomologique de Cotonou [CREC], Benin) provided an overview of the distribution, abundance and biting behavior of *Aedes aegypti* and *Aedes albopictus* in Benin to assess arbovirus transmission risks and inform control strategies. Through a combination of human landing catches, ovitraps and dipping techniques, the study revealed that *Ae. aegypti* is widely distributed across the country, while *Ae. albopictus* is confined to the southern regions. Both species exhibited exophagic behavior, with peak biting activity observed at dawn and dusk. The research identified domestic containers and used tires as the primary breeding sites for both mosquito species. Co-occurrence of *Ae. aegypti* and *Ae. albopictus* was noted in certain areas, alongside sylvatic species such as *Aedes luteocephalus* and *Aedes vittatus*. This study provides the first comprehensive data on the distribution of *Ae. albopictus* in Benin, highlighting key areas for arboviral disease preparedness and targeted vector control interventions.

David P. Tchouassi (International Centre of Insect Physiology and Ecology [ICIPE], Kenya) delivered a presentation on the behavioral responses of *Ae. aegypti* and *An. gambiae* mosquitoes to plant- and vertebrate-derived compounds, emphasizing sex- and species-specific sensitivities. While male mosquitoes are exclusive plant feeders, females of both species exhibited stronger attractive responses to certain plant-derived odors. Conversely, males demonstrated notable attraction to certain vertebrate-derived cues, suggesting potential roles in enhancing mating competitiveness. These findings reveal opportunities to exploit highly attractive cues for both sexes, particularly for male-targeted interventions. In the context of sterile insect technique (SIT) programs, where the release of sterile males aims to suppress mosquito populations, understanding male ecology and optimizing their mating success are critical for program efficacy. This work underscores the importance of integrating behavioral ecology into vector management, paving the way for innovative tools to combat mosquito-borne diseases.

Sperancia C. Lushasi (IHI, Tanzania) presented findings on interspecific competition between *Ae. aegypti* and three major African *Anopheles *vector of malaria (*An. arabiensis*, *An. gambiae* sensu stricto [*An. gambiae* s.s.] and *An. funestus*) at the larval stage in semi-field settings. Results revealed that *Ae. aegypti* outcompeted *Anopheles* species, with the former having higher survival rates and greater tolerance to resource-limited conditions. However, competition significantly affected both genera, reducing survival rates, delaying development and producing smaller adults in mixed-species populations compared to single-species populations. Cannibalism and predation varied depending on habitat size and food availability. These findings emphasize the role of interspecific competition in shaping mosquito traits that influence vectorial capacity, providing critical insights for predicting disease transmission dynamics.

Ana C. Bahia (Universidade Federal do Rio de Janeiro [UFRJ], Brazil) presented pioneering research on the fungal microbiota (mycobiome) of *Ae. aegypti*, exploring its potential role in arbovirus transmission. While bacterial influences on mosquito-borne virus transmission are well-documented, the role of fungi remains underexplored [5]. In the study reported, the fungal community was found to increase throughout the mosquito's development, with differences in diversity and abundance observed between laboratory-reared and field-collected mosquitoes, and also influenced by the presence of *Wolbachia*. Fifty-three fungal isolates were identified, including *Candida guilliermondii*, which exhibited significant antiviral activity against Zika virus in in vitro experiments. Metabolite analysis revealed potential fungal molecules with inhibitory effects on arbovirus infection, paving the way for further testing. These findings highlight the potential of targeting mosquito-fungus-virus interactions to develop novel strategies for blocking arbovirus transmission.

Roch K. Dabiré (Institut de Recherche en Sciences de la Santé [IRSS], Burkina Faso) presented findings from longitudinal entomological surveys conducted from September 2021 to July 2024 in three sites of Bobo-Dioulasso, monitoring *Ae. aegypti* populations as part of a pilot SIT intervention. Among the collected adult mosquitoes, *Culex quinquefasciatus* (> 80% of catch) and *Ae. aegypti* (approx. 15% of catch) were the most abundant adult mosquitoes, with *Aedes* populations peaking during the rainy season (May–November), coinciding with malaria vector proliferation. Larval and pupal collections revealed *Culex* (> 70%) and *Aedes* (> 20%) dominance.* Stegomyia* indices (i.e. entomological indicators used to measure the presence and density of *Aedes* mosquitoes) exceeded arbovirus outbreak risk thresholds, suggesting the urgency for enhanced surveillance.

Jacklin F. Mosha (National Institute for Medical Research [NIMR], Tanzania) presented a comprehensive study on the bionomics, arbovirus infection rates and insecticide susceptibility of *Ae. aegypti* mosquitoes in Tanzania's Lake Zone across urban, semi-urban and rural settings. Additionally, the study assessed human exposure to dengue virus through serological surveys using rapid diagnostic tests (RDTs) and enzyme-linked immunosorbent assays. *Aedes aegypti* was the only *Aedes* species identified during a 4-month survey (October–January 2023). Used tires and discarded containers were the most common and productive habitats, particularly in urban areas, which also exhibited the highest container index (57.4%) and dengue exposure. Among surveyed individuals, 5.8% were RDT-positive for dengue, and 25% showed evidence of past infections. The adult mosquitoes exhibited resistance to commonly used insecticides, including deltamethrin, permethrin and alpha-cypermethrin, but remained susceptible to pirimiphos-methyl and bendiocarb.

Ademir J. Martins (Fundação Oswaldo Cruz [Fiocruz], Brazil) described the results from insecticide resistance monitoring for *Ae. aegypti* in Brazil, which has been a key component of chemical control surveillance since the late 1990 s. The MoReNAa network has been monitoring 70–120 mosquito populations biannually, with data collected up to 2013. Based on the results of WHO-standard bioassays and dose–response tests, the study identified widespread resistance to key insecticides, notably the larvicide temephos and the adulticide pyrethroids, with emerging resistance to malathion. Several resistance mechanisms, including the expansion of knockdown resistance (kdr) mutations and metabolic resistance, were characterized through enzymatic assays and genotyping techniques. kdr is a well-characterized mechanism of resistance to pyrethroid insecticides in many insect species. Recent assessments of resistance to newer insecticides, including the larvicide spinosad and combined pyrethroid formulations for adults, showed that *Aedes* populations remain susceptible to these compounds, offering hope for more effective control tools. This research underscores the critical need for a multifaceted approach to vector control, incorporating new insecticides, alternative control methods and ongoing surveillance to address the growing challenge of insecticide resistance in *Ae. aegypti* populations.

Filémon T. Tokponnon (CREC, Benin) also reported on the susceptibility of adult *Ae. aegypti* to insecticides used in public health programs in Benin and examined resistance mechanisms. CDC bottle bioassays revealed resistance to permethrin and deltamethrin, while the mosquitoes showed complete susceptibility to bendiocarb. Biochemical assays revealed elevated glutathione S-transferase activity and reduced α and β esterase activity in field populations compared to the susceptible Rockefeller strain, with no significant differences in mixed-function oxidases. Genotyping identified three kdr mutations (F1534 C, S989P and V1016G) at high frequencies, with 80.9% of mosquitoes carrying at least one mutation and 13 individuals exhibiting all three. Notably, this is the first report of the V1016G mutation in Africa and the first time all three kdr mutations have been detected in *Ae. aegypti* in Benin. The presence of the V1016G mutation in West Africa marks a significant geographic expansion of this mutation and represents an important finding. These results highlight widespread pyrethroid resistance, enhanced detoxification enzyme activity, and prevalent kdr mutations, underscoring the urgent need for alternative vector control strategies in Benin.

Athanase Badolo (University Joseph Ki-Zerbo [UJKZ], Burkina Faso) reviewed the ecology of *Ae. aegypti* in Burkina Faso, focusing on preferred breeding sites, resting behavior and the presence of kdr mutations, while also presenting findings on its biting patterns along an urbanization gradient. Over a 3-month period during the rainy season, biting activity was monitored using human landing catches in urban, peri-urban and rural areas, with continuous data collection over 48-h periods both indoors and outdoors. A total of 4697 mosquitoes were captured, with biting rates peaking at 1000 bites per person every 48 h outdoors in urban areas, dropping to 100 bites per person every 48 h indoors. Contrary to the belief that *Ae. aegypti* is strictly diurnal, biting occurred throughout the 24-h period with two peaks: 5–9 a.m. and 4–7 p.m. Along the urban-to-rural gradient, biting rates declined, and the morning peak disappeared in rural areas, while the evening peak remained consistent. Further studies are underway to explore potential links between genomic variants and these behavioral differences.

A turbo-talk session was then organized, providing eight participants the opportunity to present their posters in a concise and dynamic format. Participating in the turbo-talks sessions were, among others: Olukayode G. Odufuwa (IHI, Tanzania), who presented “House modifications using insecticide treated screening of eave and window as vector control tool: evidence from a semi-field system in Tanzania and simulated epidemiological impact”; Fabien Z. Vulu (University of Kinshasa (UNIKIN), Democratic Republic of Congo), who presented “Geographic expansion of the introduced *Aedes albopictus* and other native *Aedes* species in the Democratic Republic of the Congo”; Borel T. Djiappi (Dschang University, Cameroon), who presented “*Aedes* mosquito distribution along a transect from rural to urban settings in Yaoundé, Cameroon”; Sare D. Zoulkifilou (École polytechnique d'Abomey-Calavi—University Abomey Calavi, Benin), who presented “Insecticide resistance of *Aedes aegypti* mosquitoes: co-occurrence of multiple kdr mutations in the Abomey-Calavi district”; and Julien B. Zahouli (Centre Suisse de Recherches Scientifiques [CSRS], Côte d’Ivoire), who presented “Evaluation of *Aedes aegypti* larval indices and eco-bio-social determinants and Fludora® Co-Max efficacy against insecticide-resistant adult populations in Côte d’Ivoire.”

## Session 2: integrated approaches to vector surveillance

Two keynote speakers were invited to open the session on integrated approaches to vector control, Javier Lezaun and Marta Maia.

In his address to the assembly, Javier Lezaun (University of Oxford, UK) highlighted the challenges of community engagement in the control of mosquito-borne diseases, a cornerstone of the integrated strategy advocated by the WHO [6]. Communities are typically invited to participate in interventions through direct actions (e.g. elimination of mosquito breeding sites) or awareness campaigns (e.g. adoption of improved practices). However, these efforts are often not self-sustaining and are designed primarily to validate specific interventions. Furthermore, they are rarely integrated into broader policy frameworks. Emerging movements, especially during epidemics, demonstrate the presence of “uninvited” participation in which communities mobilize against interventions that fail to meet their expectations. For example, during the summer of 2024 in Spain, a significant outbreak of West Nile virus resulted in numerous human cases and fatalities, sparking widespread public protests against the government. This form of community engagement requires careful consideration, deeper understanding and proper evaluation. Encouraging synergies between “invited” and “uninvited” participation could foster the adoption of new practices, technologies (e.g. mobile apps such as Mosquito Alert), personal protection measures (e.g. repellents), mosquito-release methods and regulatory changes (e.g. the registration of *Wolbachia* as a pesticide in the USA).

Marta Maia (Kenya Medical Research Institute [KEMRI], Kenya) emphasized the importance of developing effective, low-cost tools like MALDI-TOF MS (matrix-assisted laser desorption/ionization coupled to time-of-flight mass spectrometry) for vector discrimination. This technology improves understanding of disease vectors by enabling the rapid identification of mosquito species [7] and determination of critical entomological parameters, such as blood meal sources [8], parity and infection status. The method enables the creation of libraries for the identification of adult and larval stages of major Afrotropical malaria vectors, allowing important information to be shared via an open-access platform. This speaker presented results using this method, including a high accuracy in species identification for adult mosquitoes (97%) and mosquito larvae (95%), blood meal source identification (96.1%) and parity (*An. gambiae*: specificity 63%, sensitivity 91%; *An. funestus*: specificity 80%, sensitivity 90%). This speaker concluded by showcasing a data-sharing platform under development, which could be integrated with National Malaria Control Programs. The widespread availability of MALDI-TOF MS instruments in microbiology laboratories across sub-Saharan Africa opens new opportunities for rapid, cost-effective and enhanced malaria vector surveillance.

Following these lectures, nine participants were invited to share the results of their studies using integrated approaches in their projects on vector-borne disease control.

Bachirou Tinto (Institut National de Santé Publique [INSP], Burkina Faso) presented the “ArboFaso” project, which aims to enhance the control of arboviral diseases in Burkina Faso through an integrated approach encompassing studies on humans, domestic animals (e.g. horses, poultry), wild animals (e.g. birds, bats) and mosquitoes [9]. The project spans 3 years (December 2023 to November 2026) and will be implemented in urban and semi-urban areas. The study aims to provide detailed insights into circulating arboviruses, assess the immunization status of the population, examine the role of animals as potential reservoirs and sentinels and identify the mosquito vectors involved in transmission. Based on the results of the project, a risk map will be developed to guide health authorities and a technical report will be produced that includes recommendations to improve arboviral surveillance and control, which will be submitted to the Ministry of Health.

Debora C. Kajeguka (Kilimanjaro Christian Medical University College [KCMUCo], Tanzania), highlighted the potential of using antibody responses to *Aedes* salivary proteins as a tool to measure exposure to *Aedes* mosquito bites in Northern Tanzania. The *Aedes* Nterm-34 kDa salivary peptide has been used in various settings to assess the risk of arboviral infections such as dengue, Zika and chikungunya [10]. The study, which was conducted in three villages in Bondo, Tanga region (Tanzania) across different seasons (rainy, dry and short rainy) between 2021 and 2022, analyzed blood samples using ELISA tests. The results revealed significant seasonal variations in antibody levels, with higher levels of anti-peptide immunoglobulin G (IgG) antibodies observed in dengue-positive individuals compared to dengue-negative ones. The findings suggest that anti-Nterm-34 kDa IgG antibodies are a reliable indicator of human exposure to *Aedes* mosquito bites and could serve as a biomarker for assessing the risk of arboviral infections. These insights may guide more targeted public health interventions as arboviral diseases continue to spread.

The presentation of Richard Malumba (Makerere University, Uganda) aimed to demonstrate how innovative artificial intelligence (AI) solutions can be used to improve the surveillance of vector-borne diseases. In Uganda, 27–34% of all outpatient consultations and 19–30% of all hospital admissions are malaria-related; yet malaria is often not diagnosed in time and one third of diagnoses are incorrect. The aim of the “Ocular” project is to develop learning approaches for the microscopic diagnosis of malaria. The project is in the pilot phase in four regional referral hospitals and two district hospitals. Around 6000 images of thick blood smears (to detect the presence of parasites) and thin blood smears (to identify plasmodial species) have been collected. Laboratory technicians have been trained and a monitoring framework has been set up to automate the collection, analysis and presentation of data, meeting the needs of health centers at the national level. In the future, the project aims to leverage data sets from other sources (registries, project data, social media, vector and environmental data) to predict and track outbreaks in real-time.

Luis F. Lopes (Universidade Nova de Lisboa [UNL], Portugal) highlighted the potential of citizen science as an innovative approach to mobilize communities for vector control [11]. The Mosquito Alert mobile app enables citizens to report mosquitoes, mosquito bites and mosquito breeding sites; these data support surveillance programs on the presence of invasive *Aedes* species in Europe. The INOVEC project aims to evaluate the app's effectiveness and acceptability in regions with endemic arbovirus transmission, such as Tanzania and Burkina Faso. Before deploying Mosquito Alert in the field, a stakeholder analysis was conducted to identify potential collaborators. Stakeholders in both Tanzania and Burkina Faso were classified based on their administrative level, the type and extent of their involvement, the timing of their involvement, their potential impact, their presumed interest and their influence. The analysis revealed that some stakeholders were identified as critical for obtaining necessary approvals, others for supporting field trials and yet others for utilization of the collected data. These insights will guide pilot trials in Tanzania, with the aim to improve arboviral disease control and monitor mosquito population dynamics, including the potential arrival of the invasive *An. stephensi*.

Tobias Suter (Swiss Tropical and Public Health Institute (Swiss TPH), Switzerland) reported on their exploration of the potential of using dogs to detect breeding sites of the invasive mosquito *Ae. albopictus*. The study involved training six detection dogs to differentiate the odor of water containing *Ae. albopictus* larvae from other types of water. An automated training system presented the dogs with seven holes containing various odors, including water with *Ae. albopictus* larvae, water with other larvae (*Culex* sp. and *Aedes japonicus*) and water with no larvae. The training was conducted in three progressive stages, with the final stage designed to mimic field conditions. The dogs demonstrated varying success rates, ranging from 55.6% to 84.7%, highlighting differences in individual performance. The results of the study led the authors to conclude that while detection dogs show promise in identifying *Ae. albopictus* larvae under controlled conditions, additional training in real-world environments is needed to enhance their reliability and effectiveness for field applications.

José B. Lima (Fiocruz, Brazil) described and discussed the various challenges in implementing entomological surveillance in Brazil. Currently, surveillance is conducted through applying the LIRAa (Rapid Index Survey for *Aedes aegypti*); however, several operational difficulties have been encountered. To address these challenges, the Ministry of Health has launched a pilot project involving a network of ovitraps to collect mosquito eggs in 327 municipalities. Dengue control agents and community members have been trained and sensitized to ensure the proper installation and monitoring of the traps. The data collected on vector presence and abundance are integrated into a centralized platform to generate heat maps, thereby identifying highly infested zones to guide vector control strategies. This initiative could be adapted for use in African settings to improve current understanding of mosquito population dynamics—particularly those of domestic *Aedes* species—and to assist municipalities in implementing targeted vector control measures.

Márcia F. Lenzi (Fiocruz, Brazil) described advances in knowledge on community-based vector surveillance in Rio de Janeiro’s favelas. In 2024, Brazil experienced its worst dengue epidemic in 40 years, with over 5 million cases and 3000 deaths reported by May 2024. Favelas, home to 16 million people in the country, are particularly vulnerable to dengue and *Ae. aegypti* mosquitoes due to persistent social and environmental challenges, such as inadequate sanitation, restricted access and high levels of violence. The “Entomofavela” project was launched in Maré, a favela with 140,000 residents, to engage local youth in vector control efforts. Trained by the Oswaldo Cruz Institute, these young participants carry out activities such as mapping areas, deploying ovitraps, identifying mosquito species, surveying vector density, and analyzing environmental factors influencing mosquito reproduction. The project also evaluates the effectiveness of community agents in controlling mosquito populations and works with local organizations to design and implement tailored control messages (Additional file 1: Figure S1). These efforts take into account the unique context of favelas.

Pie Müller (Swiss TPH, Switzerland) presented a citizen science-based intervention to combat dengue in the Cocody-Bingerville health district of Abidjan, Côte d’Ivoire, a district which has reported the majority of dengue cases in the country since 2017. This innovative approach, which actively engaged local residents, tackled the challenges of larval control with a focus on transient *Aedes* breeding sites, such as discarded plastic containers and tires, habitats often overlooked by spatial adulticide spraying. To lay the fieldwork, a population survey was conducted in two neighborhoods of Cocody-Bingerville to gather demographic information and assess awareness of dengue. The survey identified discarded containers and tires as the primary types of larval breeding sites. Subsequently, the local population was invited to co-design the intervention alongside the research team and received training in managing breeding sites. The intervention will be evaluated through a randomized controlled trial with four study arms: a control arm with no intervention; an arm focused solely on larval habitat elimination; an arm using ovitraps (BG-GAT); and a combined arm implementing both larval habitat elimination and BG-GAT. A final workshop to engage the local population and review outcomes is planned at the conclusion of the trial.

## Session 3: innovation in vector control

This plenary session was initiated with two keynote lectures aimed at reviewing evidence of innovative control strategies and tools for improving *Aedes* control.

**Fig. 1:**
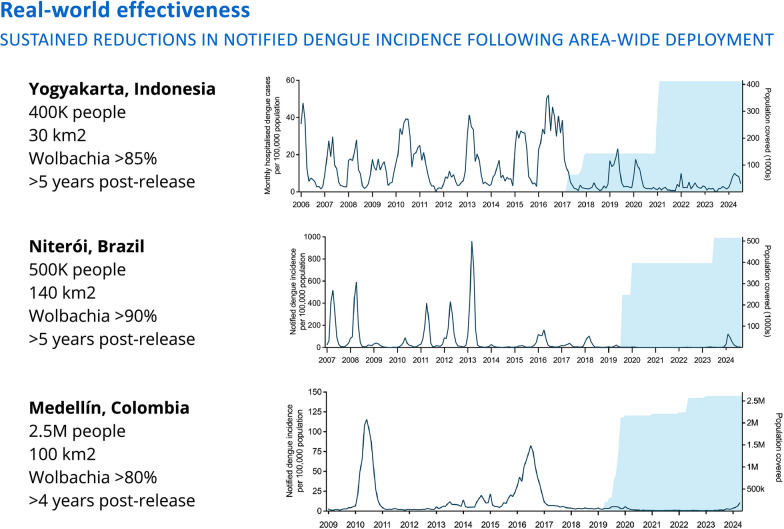
Reductions in the incidence of reported dengue cases after deployment of *Wolbachia*-infected *Aedes* in Indonesia, Brazil and Colombia Courtesy of Jeremie Gilles, World Mosquito Program, France

Jérémie Gilles (World Mosquito Program [WMP], France) highlighted recent advances and ongoing challenges in scaling up the *Wolbachia* method to prevent dengue outbreaks in endemic regions. *Wolbachia* is a naturally occurring bacterium used as a biological control agent to block the transmission of arboviruses. When introduced into mosquito populations, *Wolbachia* can inhibit the spread of mosquito-borne diseases such as dengue, Zika and chikungunya [12]. The WMP's *Wolbachia*-based approach has proven pivotal in combating ABDs and safeguarding communities worldwide. Over the past decade, this method has been deployed in 14 countries, providing protection to more than 13.3 million people, with its effectiveness for dengue control validated by multiple field trials [13] (Fig. 1). Recent data from both the Aburrá Valley in Colombia and from Niterói, the first Brazilian city to be fully protected using the *Wolbachia* approach, reveal the lowest reported dengue incidence in more than 20 years [14]. Based on these findings, the WHO Vector Control Advisory Group (VCAG) concluded in 2020 that *Wolbachia*-infected *Ae. aegypti* mosquitoes have demonstrated clear public health benefits in the fight against dengue.

Jennifer Stevenson (Swiss TPH, Tanzania) then reviewed the “Vector Control Pipeline for *Aedes* from the Pre-20 th Century Practices to future Innovations.” She first reminded the audience that significant control efforts in the Americas using continent-wide campaigns of larval oiling and DDT (dichlorodiphenyltrichloroethane) were successful in eradicating *Ae. aegypti* from 22 South American countries in the early 1960 s. However, reduced political support and insecticide resistance led to re-invasion. New tools for controlling *Aedes* mosquitoes were consistently introduced in the late 20 th and early 21 st centuries that integrated various methodologies, such as genetic and biological control methods. To date, *Wolbachia* for population replacement [15] and spatial repellents [16] have proved efficient/effective strategies for reducing dengue incidence in various epidemiological settings. However, robust evaluations of *Aedes* control measures in Africa remain limited, despite the origin of the vector within the continent and its current significant arboviral burden. Generating evidence and adapting control strategies to local contexts are essential for combating ABDs in the region.

Following the keynote lectures, the session included 10 presentations that focused on specific developments in vector control.

Audrey Lenhart (Centers for Disease Control and Prevention [CDC], USA) provided an overview of innovative *Aedes* tools under review by the VCAG. The VCAG assesses the public health value of new interventions and provides guidance on developing the necessary evidence base to support these assessments. For *Aedes*, the products under evaluation are chemosensory interferences (e.g. spatial repellents with transfluthrin), vector traps (including adulticidal oviposition and auto dissemination devices), reduced pathogen transmission through microorganism (i.e. *Wolbachia*), and sterilization agents (i.e. SIT combined with *Wolbachia*). Among the multiple *Aedes*-targeted products that are on the WHO evaluation pathway, the *Wolbachia*-infected *Ae. aegypti* (wMel) product is nearing the final guideline stage.

Amy C. Morrison (University of California Davis (UC Davis), USA) gave an overview of the efficacy of a spatial repellent for controlling *Aedes*-borne virus transmission in Iquitos, Peru. A cluster-randomized controlled trial was conducted to assess the impact of a transfluthrin-based spatial repellent on human arboviral infection and on *Aedes* populations. The results from 2907 households across 26 clusters (1578 participants) showed that the spatial repellent significantly reduced *Aedes*-borne virus infection by 34%, and reduced indoor *Ae. aegypti* female mosquito abundance by 28.6%, compared to a placebo [16]. A second randomized controlled trial is currently underway in Colombo, Sri Lanka.

Jeremy Bouyer (Centre de coopération internationale en recherche agronomique pour le développement [CIRAD], France) gave an overview of the progress of the SIT and boosted SIT approaches against *Aedes* invasive species worldwide. The boosted SIT is a variant of the SIT in which sterile males are also used as vectors of biocides (e.g. pyriproxyfen) to target females and larval sites. This approach can be used at the beginning of a suppression effort, either alongside the SIT or with other interventions [17]. To date, there are 36 on-going SIT-related field projects, including 30 involving the SIT, two with the boosted SIT with pyriproxyfen and two with the SIT combined with the incompatible insect technique. Jeremy Bouyer indicated that the SIT and particularly the boosted SIT can effectively suppress *Aedes* populations in various settings, achieving reductions ranging from 50% to 90% in adult density and even reducing dengue incidence [18].

Bart Knols (K&S Consulting, Netherlands) gave a presentation on the efficacy/effectiveness of mass trapping methods of arboviral vectors conducted in the Maldives and the Philippines. The deployment of mosquito traps (BG-Mosquitaire CO_2_ traps) baited with human odors, used in combination with measures aimed at controlling mosquito larvae in breeding sites, resulted in the successful elimination of mosquito populations on some islands and a reduction in mosquito populations of over 90% in other settings [19]. The use of mosquito traps offers an affordable and environmentally friendly approach to manage mosquito-borne disease risks on small tropical islands.

The second session opened with a presentation of Rosemary Lees (Liverpool School of Tropical Medicine—Innovation to Impact [LSTM-I2I], UK) who addressed the need for enhanced methodologies in mosquito control to generate more reliable data. To facilitate the effective choice, use and monitoring of new tools, it is crucial that appropriate methods are validated and widely available to measure efficacy with sufficient accuracy, sensitivity and reproducibility [20]. The Innovation to Impact (I2I) initiative developed a standardized framework and an online “Methods Landscape” to guide the vector control community in selecting optimal methods for generating relevant data. This tool prioritizes context, endpoints and cost, focusing initially on insecticide-treated nets and indoor residual spraying against malaria vectors. It also emphasizes the need for further research and method development, with plans to expand to *Aedes* vector surveillance and control.

Frédéric Schmitt (Envu, France) described the iDEM Trial that was implemented in urban Malaysia to measure the effectiveness of integrated vector management, including outdoor residual spraying, auto dissemination traps and active community engagement on the incidence of dengue compared to routine vector control actions. This randomized controlled trial conducted in 280 localities of Kuala Lumpur and Putrajaya (involving a total of 734,900 people) showed only a 14% decrease in dengue incidence in the intervention arm compared to the control. However, post-hoc analysis showed a 29% and 43% decrease in the number of dengue cases during outbreaks and in hotspot clusters, respectively, in the intervention arm. The results of this study demonstrated the public health benefit of integrated vector management for dengue control.

Augustino T. Mmbaga (IHI, Tanzania) reviewed the efficacy of autodissemination devices using pyriproxyfen for malaria vector control in both rural and urban Africa. The autodissemination technology, which transfers lethal concentrations of chemical insecticides, such as pyriproxyfen, to the oviposition sites of mosquitoes, capitalizes on the resting behavior of gravid mosquitoes and results in reduced adult emergence. Several trials have demonstrated the potential of autodissemination with pyriproxyfen to reduce the densities of malaria vectors [21]. Key determining factors for the successful introduction of this approach include the development of scalable autodissemination devices, optimized pyriproxyfen formulations, assessment of its integration with existing conventional larviciding methods and community acceptance.

Gerry F. Killeen (University College Cork [UCC], Ireland) presented findings from entomological and sociological assessments of hessian fabric transfluthrin vapor emanators for protection against *Aedes* mosquitoes in urban Tanzania and Haiti. In semi-field trials conducted in Dar es Salaam, Tanzania, human landing rates of *Ae. aegypti* were reduced by 50–60% [22]; in contrast, negligible human landing rate reduction was observed in Port-au-Prince, Haiti [23]. Despite this mixed result, many community users in Haiti who received the devices expressed moderate to high satisfaction with their protection against indoor mosquitoes [24]. Further research is needed to understand the discrepancy between sociological perceptions and entomological outcomes and to evaluate the efficacy of these emanators in preventing arbovirus infections.

The subsequent presentation by Tullu Bukhari (KEMRI, Kenya) addressed the community acceptance and the potential of the* Anopheles* symbiont *Microsporidia MB* for controlling *Plasmodium* transmission by *An. stephensi* in Ethiopia. *Microsporidia MB*, a naturally occurring endosymbiont in *Anopheles* mosquitoes, inhibits *Plasmodium* transmission and represents a promising candidate for a transmission-blocking strategy that may involve mosquito release. The findings from social surveys conducted in Ahero, Western Kenya, indicated strong community support, with 80% of household heads (*n* = 8000) expressing willingness to accept and participate in a *Microsporidia MB*-based mosquito release strategy. Acceptance levels were influenced by various factors, including community context, perceived disease risk, gender, education, malaria knowledge and prior experience with the disease [25].

Paulo E.M. Ribolla (Universidade Estadual Paulista Júlio de Mesquita Filho [UNESP], Brazil) concluded the session with a presentation on developing bioinsecticides based on RNA interference (RNAi) to control *Aedes* mosquitoes. RNAi molecules, such as short-hairpin RNAs (shRNAs), are designed to disrupt the expression of vital mosquito genes, leading to their death [26]. This speaker outlined the production pipeline, including target selection, plasmid construction, yeast transformation and bioinsecticide formulation. Laboratory assays showed that consuming SEMA1 A yeast caused significant mortality (60%) in *Aedes* larvae within 10 days. Future work will focus on nanoencapsulating RNAi constructs and scaling up yeast-based shRNA production for practical mosquito control applications.

## Industry session: developing public–private partnership for success

The focus of this session was the latest advances in vector control tools and technologies, and featured private-sector representatives from agrochemical companies (Clarke, Envu, SC Johnson) and R&D firms (Biogents AG, Yorkool, Vestergaard Frandsen).

Rosemary Lees (Liverpool School of Tropical Medicine [LSTM], UK) opened the session with an overview of the Vector Control Professional Placement Programme (VCP^3^), funded by the Gates Foundation. This program aims to identify and place qualified researchers from African institutions within established manufacturers of vector control products/solutions for hands-on experience in product development, from design to launch. The 12-month placements provide training in areas such as business planning, quality assurance, regulatory affairs and networking, with applications that opened on the Innovation to Impact—VCP^3^ website in January 2025.

Laetitia Leroy (Clarke, USA) presented Clarke's innovative and integrated vector control solutions for dengue and malaria management. After providing a review of Clarke's portfolio, she shared efficacy data on natural products such as Qalcova® and Natular G30; Natular® DT contains spinosad, a bioinsecticide derived from the bacterium *Saccharopolyspora spinosa*). Spinosad offers a unique mode of action for managing insecticide resistance, supports various habitat applications and has a favorable environmental profile. Additionally, it is a promising tool for targeting *An. stephensi*, the invasive urban malaria vector that is spreading in Africa.

**Fig. 2:**
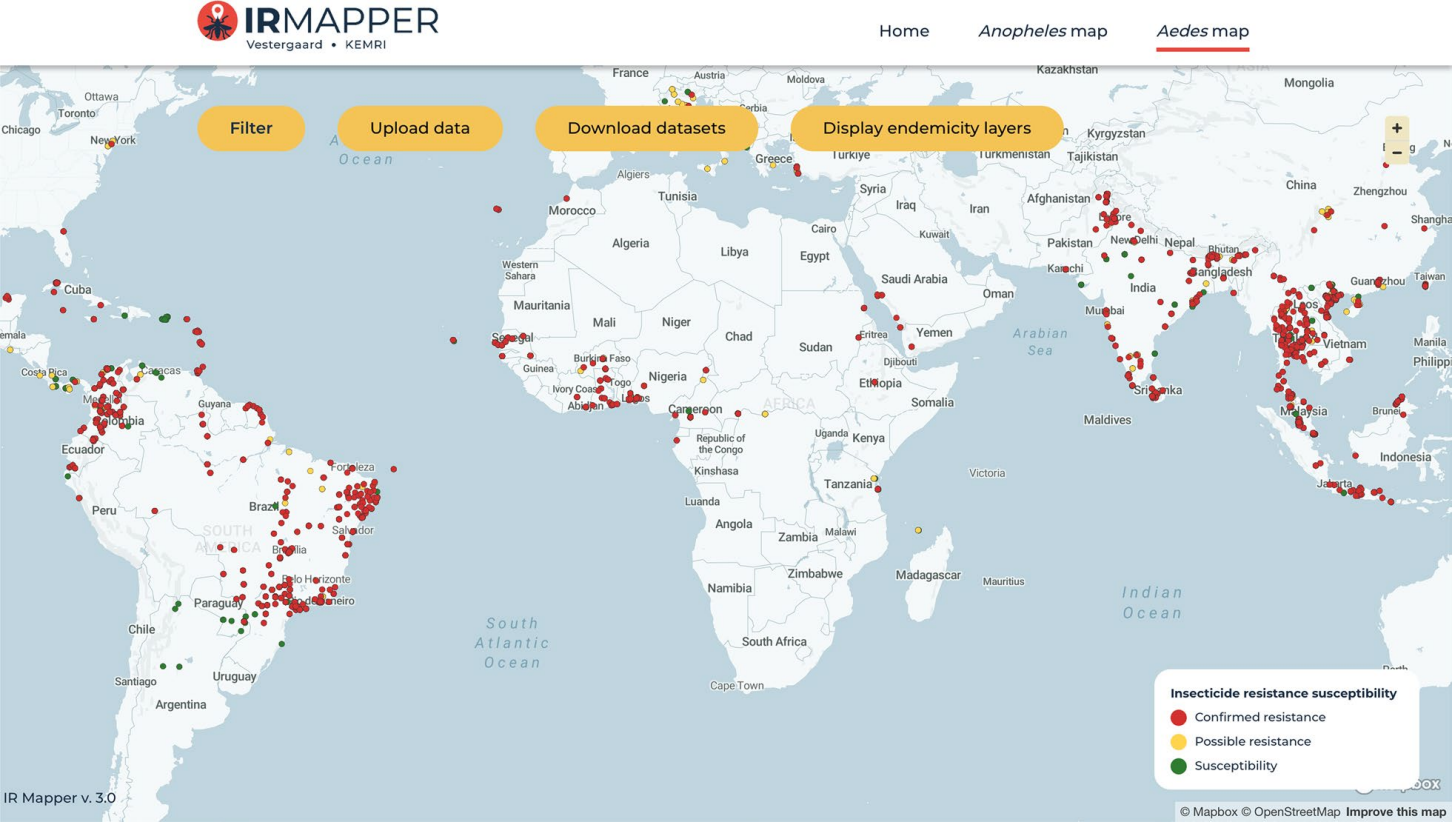
Global distribution of insecticide resistance in *Aedes aegypti* and *Aedes albopictus*, visualized using IR Mapper (https://www.irmapper.com/). The maps are freely accessible from IR Mapper at https://www.irmapper.com/ (accessed 29 April)

Duncan K. Athinya (Vestergaard Frandsen, Kenya) presented the IR Mapper platform, which visualizes spatial–temporal trends in insecticide resistance in mosquitoes. Initially developed for malaria vectors, the platform now includes data on *Ae. aegypti* and *Ae. albopictus* (Fig. 2). As of July 2024, the platform features over 10,000 unique data fields, with the majority focused on *Ae. aegypti* in the Americas (59%) and *Ae. albopictus* in Asia (83%); Africa only accounts for a small percentage (6% of total), highlighting the need for more insecticide resistance studies as *Aedes* species expand across the continent.

Thomas Mascari (SC Johnson, USA) reviewed SC Johnson's spatial repellent programs developed under the SC Johnson Healthier World Initiative. Products like Guardian™ and Mosquito Shield™ have shown potential in reducing the risk of mosquito-borne diseases such as malaria, dengue and leishmaniasis. Clinical trials have demonstrated the effectiveness of these products, supporting potential policy recommendations from WHO and United Nations Children's Fund (UNICEF) [16, 27]. These spatial repellents have been implemented in real-world and humanitarian settings across 17 countries, with emergency use authorization granted by the U.S. Environmental Protection Agency for dengue control in Puerto Rico.

Astrid Schuhbauer (Biogents AG, Germany) presented findings from *Ae. albopictus* monitoring in Fürth, Germany, using BG-GAT (Gravid* Aedes* Trap) and BG-Pro (professional modular trap system) traps with CO_2_ and BG-Lure attractants. Monitoring revealed a significant tiger mosquito population, especially in gardens and residential areas. Integrated control measures, including breeding site removal,* Bacillus thuringiensis israelensis* (Bti) treatments and BG-GAT deployment, have effectively reduced mosquito populations. Trap collections provided critical data on mosquito population dynamics and can be used as integrated vector management.

Yin Qing (Yorkool, China) introduced Yorkool, a company specializing in R&D for new mosquito control tools. Since its inception in 2019, Yorkool has developed Good Laboratory Practice-certified pesticide testing facilities and created four dual-active ingredient long-lasting insecticidal nets, combining deltamethrin with piperonyl butoxide (PBO) or chlorfenapyr (Yorkool G3, G4, G5, NG). These innovations aim to improve the control of pyrethroid-resistant mosquitoes, contributing to enhanced vector management strategies.

## PhD students: promoting research and career development

The PhD session was organized to provide seven early career researchers with the opportunity to present their work.

Christophe R. Keumeni (Centre for Research in Infectious Diseases [CRID], Cameroon) presented findings on insecticide resistance in *Ae. aegypti* and *Ae. albopictus* mosquitoes from five localities in Cameroon, emphasizing phenotypic resistance and its correlation with kdr mutations. *Aedes aegypti* was found to exhibit high resistance to pyrethroids and carbamates, while *Ae. albopictus* showed moderate resistance. Both species remained fully susceptible to organophosphates. Pre-exposure to PBO partially restored mosquito mortality, implicating cytochrome P450 enzymes in the observed resistance. The study also identified the F1534 C kdr mutation, which was nearly fixed (> 96%), along with the V1016I and V410L mutations, both more prevalent in resistant mosquitoes and strongly associated with pyrethroid resistance. These findings underscore the urgent need for targeted strategies to manage insecticide resistance in Cameroon.

Frank Tenywa (IHI, Tanzania) reported on the risk of dengue transmission by *Ae. aegypti* mosquitoes across the Ilala, Kinondoni and Temeke districts in Dar es Salaam. A total of 83,530 mosquitoes were collected in a 2-year longitudinal survey employing four sampling methods, of which 7.3% were identified as *Ae. aegypti*. The highest mosquito denisity (51.5%) was recorded in Temeke district. Dengue virus serotype 2 (DENV-2) was detected in both Temeke and Kinondoni, with a higher infection rate (11.9%) recorded in Temeke. The study also revealed resistance in *Ae. aegypti* to pyrethroids and organochlorines, although the species remained susceptible to carbamates, which are recommended for control interventions. The F1534 C mutation was detected across all districts, highlighting the critical need for focused vector control measures to mitigate dengue transmission risks, particularly in high-risk areas such as Temeke.

Bertille Carine Theno Djapoum (University of Yaoundé I, Cameroon) investigated the bioecology, distribution and insecticide susceptibility of *Ae. aegypti* and *Ae. albopictus* across three ecological zones in Cameroon: Bertoua (savanna), Kribi (port city) and Sangmelima (forested area). In this study, *Ae. albopictus* was found to be the predominant mosquito species in Kribi and Sangmelima, while *Ae. aegypti* was more common in Bertoua. Tires in Sangmelima and plastic containers in Kribi were identified as the most abundant breeding sites. High* Stegomyia* indices in Kribi and Bertoua indicated a significant risk of dengue and yellow fever transmission. *Aedes aegypti* showed resistance to permethrin, deltamethrin and DDT in all sites, while *Ae. albopictus* demonstrated resistance to bendiocarb in Kribi and Bertoua. Both species remained fully susceptible to malathion. These results highlight the need for ecologically tailored vector control strategies to reduce the risk of arboviral outbreaks in Cameroon.

Frank Tenywa (IHI, Tanzania), on behalf of Silvan Hälg (Swiss TPH, Switzerland), presented a study on the serotypes and genotypes of flaviviruses and alphaviruses circulating in mosquito populations in Dar es Salaam. DENV-2 was identified in this study, and whole-genome sequencing revealed that it belonged to the cosmopolitan genotype, closely related to DENV-2 strains from Kenya and India. These findings offer valuable insights into the cross-border circulation of dengue strains and highlight the crucial role of continuous surveillance in guiding vector control strategies and public health interventions.

Sandra Ateutchia Ngouanet (Institut International d'Agriculture Tropicale [IITA], Benin) studied *Aedes* mosquito populations across three agroecosystems—palm, banana and in-house gardens—in Benin to assess species composition, arbovirus presence, *Wolbachia* symbionts and insecticide resistance. *Aedes aegypti* (93.9%) was found to be the predominant species, followed by *Ae. albopictus* (6.1%), with no arboviruses detected. Naturally occurring *Wolbachia* was found in 20% of the mosquito populations. *Aedes aegypti* exhibited complete phenotypic resistance to permethrin in two agroecosystems, linked to kdr mutations (F1534 C, V1016G/I, S989P) and overexpression of cytochrome P450 detoxification genes. This study is the first to report *Wolbachia* in *Ae. aegypti* in Benin. These findings emphasize the need for sustained vector surveillance and provide essential data to inform targeted control strategies in agroecosystems under varying insecticide selection pressures.

Temesgen Ashine (Armauer Hansen Research Institute [AHRI], Ethiopia) presented updates on the distribution and household exposure of *An. stephensi* in Ethiopia based on surveillance conducted in 26 urban areas from 2021 to 2023. *Anopheles stephensi* accounted for 7.7% of the total *Anopheles* collected, with *An. arabiensis* being the dominant species (79.5%). The study revealed that *An. stephensi* is expanding its range and prevalence in certain areas. Although *An. stephensi* exhibited zoophagic behavior, none of the collected *An. stephensi* specimens tested positive for *Plasmodium*, in contrast to *An. arabiensis*, which showed a 4.3% infection rate [28]. These findings highlight the geographic expansion of *An. stephensi* and the urgent need for enhanced entomological surveillance to assess its potential role in malaria transmission.

Borel Tchamen-Djiappi (University of Dschang, Cameroon) evaluated the efficacy of the bacterial larvicide VectoMax®G (Valent BioSciences, USA), which contains *Bacillus thuringiensis* and *Bacillus sphaericus*, in controlling *Ae. aegypti* and *Ae. albopictus* larvae under semi-natural conditions in Yaoundé. In this study, different larvicide doses were tested in tires and in plastic and metal containers; VectoMax®G was found to be highly effective, with 100% larval mortality within 24 h. Its residual effect lasted longest in tires and varied with environmental conditions. The results of this study confirm VectoMax®G as a promising tool for vector control, particularly in habitats subject to diverse environmental factors.

To recognize and reward exceptional presentations, conference participants were invited to evaluate each presenter and presentation in the PhD session based on various criteria through a questionnaire accessible via a QR code. The top three presenters, who achieved the highest scores, were Temesgen Ashine (first award), Frank Tenywa, and Borel Tchamen-Djiappi.

## Report from Round table: “Are we losing the battle against urban mosquito-borne diseases in Africa? Challenges and opportunities in controlling the spread of *Anopheles stephensi* and *Aedes* spp. mosquitoes”

The 2-h round table session was chaired by Daniel Msellemu (IHI, Tanzania) and attended by four moderators: Prosper Chacki (Executive Director of Pan-African Mosquito Control Association [PAMCA], Tanzania), Audrey Lenhart (Head of the Entomology Branch, US Centers of Disease Control and Prevention [CDC], USA), Raman Velayudhan (Unit Head, Veterinary Public Health, Vector Control and Environment Unit [VVE], WHO-NTD, Switzerland) and Jennifer Armistead (Vector Control Team Lead, US President's Malaria Initiative/US Agency for International Development [USAID], USA). The session started with two presentations designed to introduce the topic to the participants.

Samuel Dadzie (University of Ghana, Ghana) first introduced the mission of the West African *Aedes* Surveillance Network (WAASuN), which was established in 2017 to strengthen the capacity of African countries in arboviral disease surveillance and control [29]. *Aedes*-borne arboviruses are emerging as a significant threat in Africa, with recent outbreaks of dengue, yellow fever, Zika, and chikungunya reported across several West African countries [9]. A WHO-TDR (WHO Special Programme for Research and Training in Tropical Diseases) program assessment of 47 African countries revealed significant gaps in preparedness, surveillance and control capacity for arbovirus outbreaks. WAASuN addresses these shortcomings by bringing together stakeholders through meetings to discuss key topics on *Aedes* control, fostering new partnerships and organizing technical workshops to enhance capacity in *Aedes* surveillance. According to the speaker, the network has increased the surveillance capacities of its members by 76% in 6 years.

Nicodemus Govella (Population Services International, Tanzania) discussed the emerging threat of *An. stephensi* in urban Africa. Known as an urban malaria vector in South Asia and the Middle East, *An. stephensi* has recently become a major concern in Africa [30]. The adaptability of *An. stephensi* to urban environments and its ability to breed in diverse water sources heighten the risk of malaria transmission in rapidly urbanizing African cities. *Anopheles stephensi* is currently established in at least eight African countries (Djibouti, Ethiopia, Kenya, Sudan, Somalia, Nigeria, Eritrea and Ghana) and is expected to spread further. In response, WHO launched an initiative in 2022 to curb the spread of *An. stephensi* in Africa, emphasizing the need for strengthened collaboration, enhanced surveillance, improved information exchange, technical guidance and prioritized research. The surveillance and control of *An. stephensi* should be integrated with efforts targeting other malaria vectors and other vector-borne diseases, especially ABDs.

The chairperson then invited the panel of experts to discuss the factors contributing to the spread of invasive and urban mosquito vectors, and how they can be effectively addressed. According to the experts present, globalization is a key factor contributing to the spread of mosquito-borne diseases. Indeed, the total number of airline passengers doubled from just below two billion in 2000 to more than four billion in 2019, an increase that favors the spread of emerging pathogens such as arboviruses [4]. The COVID-19 pandemic is a striking example of how international transit can drive the spread of an emerging virus across the globe. The transport of goods (especially by ships carrying used tires) is known to be the primary cause for the dispersal of the tiger mosquito *Ae. albopictus* in Africa, Europe and USA [31]. Beyond that, climate change poses an increasing threat to vector-borne disease control as it can create a suitable environment for vectors and pathogens to develop and survive. However, climate change—and especially increased temperature−can contribute positively or negatively to the development of a given species. For example, elevations in temperature may be particularly suitable for the transmission of dengue as the optimal maximum temperatures for the biting rate, fecundity, development rate and immature survival of *Ae. aegypti* are higher than those for other mosquito species [32]. Support for this was provided by Raman Velayudhan who emphasized the point that *Ae. aegypti* was found surviving in water breeding sites in Southern Sudan at a temperature reaching 46 °C. Other anthropogenic factors contributing to the recent range expansion of the malaria vector *An. stephensi* and *Aedes* spp. mosquitoes include increased urbanization, inadequate infrastructure and the widespread use of water storage containers.

Daniel Msellemu also addressed the question of how national and international public health policies can be improved to better manage and control mosquito populations in urban settings. Audrey Lenhart from the US CDC noted a significant lack of integrated actions between malaria and dengue control programs globally, as well as among entomologists working on *Anopheles* and *Aedes* species. Several structural reasons contribute to this lack of interaction. Firstly, funding streams tend to be highly disease-specific, with donors allocating funds to countries based on predetermined budget programs. There is a pressing need to change how we approach “inter-disease” control; ministries and national programs should have the autonomy to advance agendas that promote multi-disease strategies and gain greater flexibility in prioritizing resource allocation. In Europe and the USA, countries have implemented mosquito control programs aimed at preventing the risk of multiple mosquito-borne diseases. In contrast, other regions, such as Africa, have historically depended on disease-specific programs, such as the National Malaria Control Program. With a few exceptions, no diseases have been eliminated through a single-disease approach. Experts advocate for a paradigm shift in vector control to achieve a multi-disease impact. One suggestion is to provide incentives for promoting integrated approaches. Additionally, the Pan American Health Organization (PAHO) has established a Global Framework to assist countries in implementing an integrated approach to control and eliminate multiple diseases [33], which could serve as a reference for developing specific guidelines for Africa. All stakeholders agree that the WHO should take a leadership role and enhance its support to countries in implementing integrated approaches. The Global Vector Control Response (GVCR) adopted at the WHO General Assembly in 2017 encourages multi-disease and intersectoral collaboration [6], although this is rarely put into practice in the field. Currently, significantly more funding is dedicated to combating malaria than to NTD, despite the rising threat of dengue and other arboviral diseases worldwide. In regions where malaria is significantly declining, a reallocation of human and financial resources towards NTD may be necessary. In Africa, dengue is often managed as an outbreak response, yet the recurrence of these outbreaks requires a more sustained and proactive approach to address the rising threat of arboviral diseases.

The experts then discussed the most promising technological and scientific approaches for large-scale control of urban mosquito populations. First, the coexistence of *An. stephensi* and *Aedes* arboviral vectors in the same urban environments presents an excellent opportunity for integrated vector management with limited resources [34]. Despite recent advances in new technologies for vector surveillance and control (see sessions 2 and 3), the experts emphasized larval source management as a powerful strategy for targeting multiple mosquito species in urban areas, while avoiding constraints related to adult feeding behavior. Larval source management has proven effective in reducing malaria incidence in Africa and Asia [35]. However, for larval source management to be effective, accurate identification of mosquito breeding sites is essential. This can be particularly challenging for *An. stephensi* and *Aedes* spp., which breed in the same man-made habitats. Ethiopia has implemented an effective surveillance program for *An. stephensi*, focusing on identifying new introductions, monitoring current distributions, tracking the spread over time and detecting common larval sites to improve preparedness and response [28]. Such a program also includes monitoring other urban mosquitoes, such as *Aedes* spp., and could easily be extended to neighboring countries. Strengthening vector surveillance by integrating AI into existing platforms offers exciting opportunities to analyze vast amounts of data, including environmental factors, and to predict and track invasive mosquitoes in real-time.

Finally, the group acknowledged the need to integrate other tools and technologies for controlling urban vectors, such as housing improvements and spatial repellents, both of which have proven effective in reducing the burden of dengue and malaria [16, 36]. Regardless of the tools or approaches used, it is essential to consider local perceptions and needs, ensuring that communities actively participate in mosquito control programs to guarantee their success. Active community participation is essential, as most mosquito breeding sites are man-made and manageable through collective efforts. This participatory approach not only would enhance the effectiveness and sustainability of interventions but also would build trust and resilience within the community, paving the way for long-term success in combating vector-borne diseases.

The following topic discussed during the roundtable was how communities and individuals can be more effectively engaged and educated to participate in mosquito control efforts. Experts emphasized the importance of decentralization, which involves delegating vector control policies/initiatives from central authorities to local levels. Identifying affected populations and understanding their perceptions, needs and level of commitments are crucial for designing and implementing locally effective strategies. Community engagement approaches should be tailored to the specific characteristics of the urban and rural communities, respectively, as these populations often differ in their exposure to diseases and access to information. Communication strategies must therefore be customized to maximize their impact. The lack of advocacy for arboviral diseases and *Aedes* control in Africa remains a significant gap, especially when compared to the strong focus on malaria, despite the growing threat of dengue epidemics. International programs such as Roll Back Malaria have been instrumental in combating malaria in Africa, and similar initiatives dedicated to ABDs should be developed to raise awareness, secure funding, and enhance efforts to tackle these diseases.

The chairman drew the session to a close by addressing how funding for mosquito control programs can be prioritized and allocated to effectively manage invasive pests, especially in resource-limited countries. Funding for NTD saw a nearly 10% decline between 2009 and 2018, amounting to a reduction of US$ 34 million (− 9.1%) [37]. This shortfall has created a significant gap in meeting program requirements, and even when funds are available, there are often substantial delays in disbursing them to countries. To maximize impact, funding should be directed toward evidence-based, locally prioritized interventions. Although evidence to inform vector control policies is largely available, it is often not accessible to local authorities who need it most. While innovative vector control technologies, such as *Wolbachia*, SIT and gene driven technologies, hold great potential, Africa is not yet prepared for their widespread adoption due to a lack of necessary skills, infrastructure and financial resources. Comprehensive guidance is urgently needed for the implementation of integrated vector management, including systematic cost assessments to enable informed decision-making. Achieving this will require robust leadership and advocacy from both national governments and international organizations. As previously mentioned, greater flexibility in reallocating funds between disease programs could enhance responsiveness to emerging challenges. Promoting a “Community of Practice” by bringing together representatives from national and local authorities, communities and non-governmental organizations could provide an excellent opportunity to identify gaps and determine the most effective strategies based on specific needs. Equally critical are efforts to strengthen capacity building, foster research and innovation and expand partnerships. These elements are key to ensuring long-term success in vector control and disease prevention. Finally, advocacy for increased, prioritized and sustained funding will be crucial to enhance vector control efforts and achieve the 2030 targets for NTD and malaria control.

## Conclusions

The International Conference on Advances in Surveillance and Control Methods for *Aedes*-Borne Diseases and Urban Vectors served as a pivotal platform for fostering scientific collaboration, exchanging knowledge, and reporting innovation in vector control. Bringing together over 200 experts from 20 countries, this event catalyzed the development of concrete strategies to combat the escalating threats posed by *Aedes* mosquitoes and invasive malaria vectors such as *An. stephensi*.

The discussions underscored the urgent need for integrated, cost-effective and scalable solutions to strengthen vector surveillance and control efforts. To effectively combat *Aedes*-borne diseases in Africa, a comprehensive and integrated approach must be prioritized. Strengthening surveillance and early warning systems is essential, as real-time data collection, predictive modeling and community-based reporting can help anticipate outbreaks and optimize response efforts. By improving vector control strategies, such as developing and expanding the use of bio-insecticides, implementing larval source management and modifying environments to eliminate breeding sites, mosquito populations can be significantly reduced using an environmentally friendly approach.

The development and deployment of novel control tools also hold great promise. Strategies such as releasing *Aedes* mosquitoes infected with *Wolbachia*, using genetically modified mosquitoes and deploying spatial repellents offer innovative and sustainable solutions for reducing disease transmission. However, successful intervention relies not only on technical solutions but also on public participation. Promoting community engagement through awareness campaigns and participatory approaches ensures that control measures are effectively adopted at the local level. At the same time, enhancing integrated vector management by coordinating multiple control strategies targeting both *Aedes* and *Anopheles* urban vectors helps maximize impact while addressing challenges like insecticide resistance.

Key actionable outcomes from the conference include:

- Enhanced collaboration: strengthening intersectoral and international partnerships to foster innovation and accelerate the implementation of new control methods
- Capacity building: expanding training programs for researchers, entomologists and public health professionals to bridge knowledge gaps in vector surveillance and control
- Policy advocacy: encouraging governments and international agencies to integrate ABD control within broader public health strategies, leveraging existing malaria and NTD programs
- Operational research & innovation: scaling up the evaluation and deployment of novel control tools, with an emphasis on locally adapted, sustainable solutions
- Community engagement: promoting participatory approaches to vector control, ensuring that interventions are culturally acceptable and actively supported by affected populations.

Moving forward, the momentum from this conference must translate into concrete initiatives, increased funding and sustained political commitment to ensure long-term success in vector control and disease prevention. With coordinated efforts and the adoption of innovative, scalable and community-driven solutions, the global health community can make significant strides in mitigating the burden of *Aedes*-borne diseases and emerging malaria threats.

## Supplementary Information

Additional file 1: Figure 1. An illustration of communication aimed at populations in favelas to combat the transmission of dengue.

## Data Availability

No datasets were generated or analysed during the current study.

## Abbreviations

ABD: *Aedes*-borne diseases
IHI: Ifakara Health Institute
IRD: Institut de Recherche pour le Développement
kdr: Knockdown resistance
NTD: Neglected tropical diseases
PBO: Piperonyl butoxide
R&D: Research and development
SIT: Sterile insect technique
RDT: Rapid diagnostic tests
VCAG: Vector Control Advisory Group
