# Supplementary figures and images for: Advances in surveillance and control methods for Aedes-borne diseases and urban vectors: report of the International Conference, August 2024, Tanzania
Source: Parasit Vectors. 2025 Jun 6;18:212. doi: 10.1186/s13071-025-06838-4 (PMC12142953; doi:10.1186/s13071-025-06838-4)

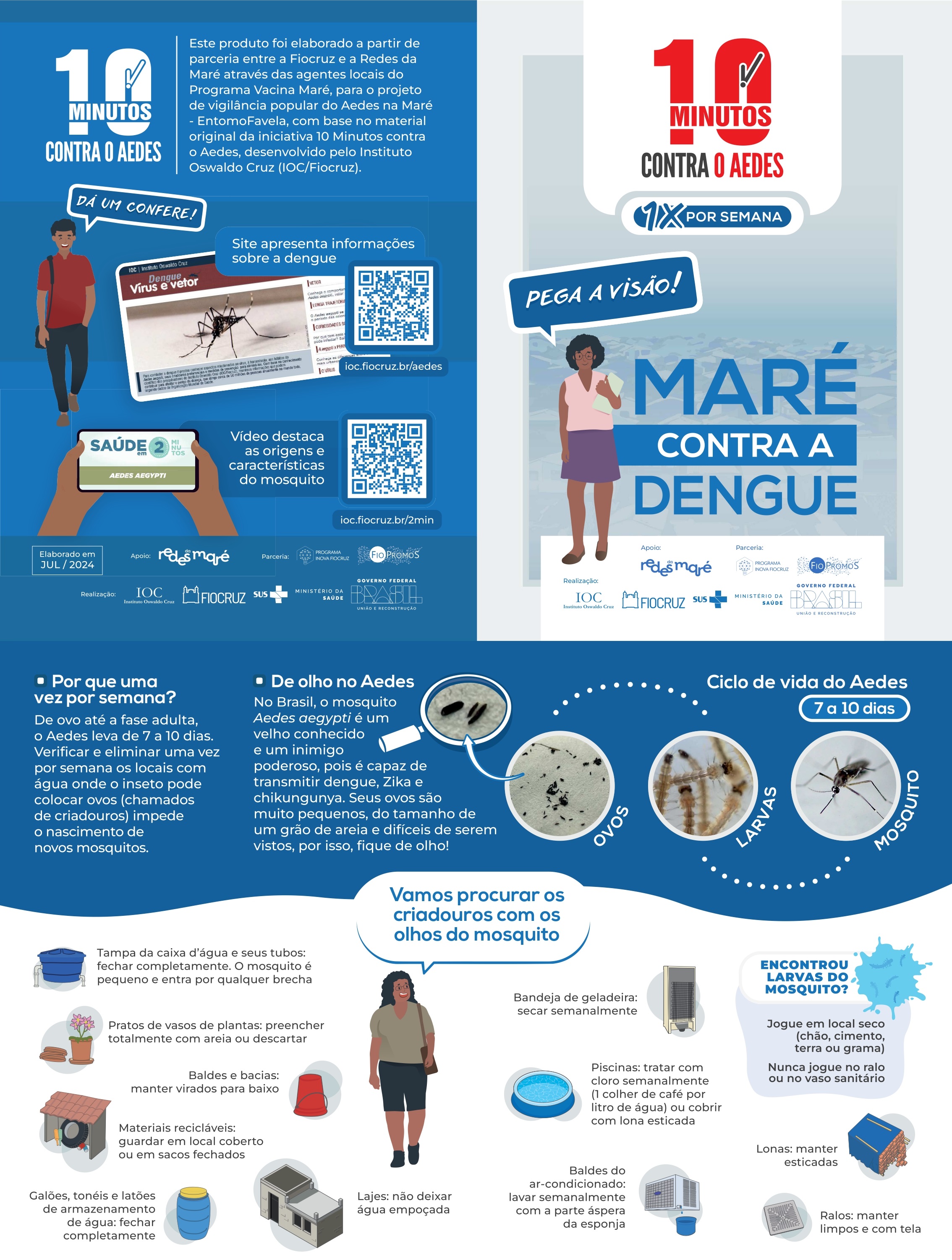

Supplement: Supplementary file 1 — Additional file 1: Figure 1. An illustration of communication aimed at populations in favelas to combat the transmission of dengue. [file 13071_2025_6838_MOESM1_ESM.jpg]
